# An Evaluation of the Effects of the Australian Food and Health Dialogue Targets on the Sodium Content of Bread, Breakfast Cereals and Processed Meats

**DOI:** 10.3390/nu6093802

**Published:** 2014-09-19

**Authors:** Helen Trevena, Bruce Neal, Elizabeth Dunford, Jason H. Y. Wu

**Affiliations:** 1 The George Institute for Global Health, The University of Sydney, P.O. Box M201 Missenden Road, Camperdown, Sydney, NSW 2050, Australia; E-Mails: bneal@georgeinstitute.org.au (B.N.); edunford@georgeinstitute.org.au (E.D.); jwu1@georgeinstitute.org.au (J.H.Y.W.); 2 The School of Public Health, Faculty of Medicine, Epidemiology and Biostatistics, Imperial College of Science, Technology and Medicine, Praed Street, Norfolk Place, London, W2 1PG, UK; 3 Royal Prince Alfred Hospital and Central Sydney Area Health Service, Missenden Road, Camperdown Sydney, NSW 2050, Australia

**Keywords:** salt reduction, food supply

## Abstract

The Australian Food and Health Dialogue set sodium reduction targets for three food categories (breads, ready-to-eat breakfast cereals and processed meats) to be achieved by December, 2013. Sodium levels for 1849 relevant packaged foods on the shelves of Australian supermarkets between 2010 and 2013 were examined. Changes in mean sodium content were assessed by linear mixed models, and the significance of differences in the proportion of products meeting targets was determined using chi-squared or McNemar’s tests. The mean sodium level of bread products fell from 454 to 415 mg/100 g (9% lower, *p* < 0.001), and the proportion reaching target rose from 42% to 67% (*p* < 0.005). The mean sodium content of breakfast cereals also fell substantially from 316 to 237 mg/100 g (25% lower, *p* < 0.001) over the study period. The decline in mean sodium content of bacon/ham/cured meats from 1215 to 1114 mg/100 g (8% lower, *p* = 0.001) was smaller, but associated with a rise in the proportion meeting the target from 28% to 47%. Declines in mean sodium content did not appreciably differ between companies that did and did not make public commitments to the targets. These data show that the Australian food industry can reduce salt levels of processed foods and provide a strong case for broadening and strengthening of the Food and Health Dialogue (FHD) process.

## 1. Introduction

Excess dietary salt causes high blood pressure [1], one of the most important risk factors for cardiovascular disease (CVD) [2]. Average adult salt intake in Australia has been estimated to be 9 g/day (~3500 mg sodium/day) [3,4], which is more than double the Australian government’s Suggested Dietary Target of 4 g/day [5]. The high level of salt intake of Australians has been estimated to cause 11% of deaths attributable to ischemic heart disease and 15% of deaths caused by stroke [6]. Thus, cost-effective policy approaches that can reduce population salt intake are urgently needed [7,8,9,10,11].

Similar to other developed countries, three-quarters of dietary salt for the Australian population comes from processed and packaged foods, rather than discretionary sources (salt added during cooking or at the table) [12]. Bread, processed meats and breakfast cereals are some of the largest contributors to salt intake in Australia, together accounting for about 20% of daily intake [13]. A systematic effort to reformulate food products to reduce their salt content is therefore a promising strategy for lowering the Australian population salt intake [9]. The United Kingdom (UK.) is considered a leader in salt reduction strategies [14], and their government-led national salt reduction program has successfully lowered population salt intake by about 15% over the last seven years to have the lowest known salt intake of any developed country [15,16]. A central pillar of the UK. policy platform is a voluntary salt reduction program that encourages food product reformulation by manufacturers to meet specific targets that are progressively lowered over time [16]. In an effort to similarly lower the salt content of processed foods in Australia, the ‘Food and Health Dialogue’ (FHD) program was launched by the Federal Government in 2009. Through a series of roundtables convened to engage key stakeholders, voluntary salt reduction targets for each of 10 food product categories and a timeline for achieving these proposed changes were established [17].

The effect of the FHD on the salt content of the food supply has so far not been quantified. A detailed evaluation of changes in the salt content in specific food categories is needed to determine the success or failure of the FHD program and to guide future policy directions [16]. The sodium reduction targets for three FHD food categories (breads, ready-to-eat breakfast cereals and processed meats) were scheduled to be achieved in December, 2013, and the aim of the present study was to assess whether these targets have been met, to quantify the magnitude of any changes in sodium content and to explore whether changes differed by manufacturer or product category. We also examined changes in product sodium content according to a manufacturer’s publicly-stated commitment to the FHD program.

## 2. Methods 

### 2.1. Product Inclusion and Exclusion Criteria

Products were included if they met the FHD definition of the three applicable food product categories [17]. Bread products were defined as being made by baking a yeast-leavened dough prepared from one or more cereal flours or meals (e.g., oat meal) and water, and examples included sliced loaf breads, rolls, bagels, English muffins and fruit breads. “Value added” products, such as cheese and bacon rolls, were excluded. Ready-to-eat breakfast cereals (referred to as breakfast cereals, hereafter) were defined as cereals with plain or mixed flakes, puffed grains, processed grains and fruit/flake mixtures with or without other ingredients. Exclusions were muesli, hot cereals and wheat biscuits. Processed meats included meat products that were ready to eat, such as luncheon meats, and cured meat products, such as bacon. Exclusions were pate, cooked uncured meats and dried meats.

### 2.2. FHD Targets and Food Company Commitment

Information on the FHD targets was derived from the FHD website [17]. We searched the food category action plans and identified the three food categories with sodium reduction targets where the dates for scheduled reductions had elapsed by January, 2014. These were maximum sodium targets applied to breads (400 mg/100 g) and processed meats, with the latter further divided into bacon/ham/cured products (1090 mg/100 g) and emulsified luncheon meats, such as mortadella or devon (spam) (830 mg/100 g). For breakfast cereals, a 15% reduction target was applied to products with more than 400 mg/100 g sodium, but there was no maximum target. There were therefore four targets for the three broad food categories defined by the FHD. FHD targets were agreed upon in May, 2010, for breads and breakfast cereals, and January, 2011, for processed meats, and scheduled to be achieved by December, 2013, for all three categories.

Food companies who had voluntarily participated and committed to the FHD targets were identified from the FHD website (the FHD participants) [17]. Collectively, the FHD participants represented the majority of the Australian market share at the time the FHD targets were formulated, producing approximately 80% of bread products, 60% of breakfast cereals and 95% of processed meats [17]. Manufacturers and grocery retailers not listed on the FHD website as having committed to the targets were described collectively as “non-participants”.

### 2.3. Data Collection

Between 2010 and 2013, data were collected from July to September of each year from the same four grocery retail stores (Coles, Woolworths, ALDI, IGA) in Sydney, Australia. Data were obtained directly from the mandatory Nutrition Information Panel (NIP), but where exactly the same product was for sale in more than one supermarket, it was recorded only once. Where the same product was presented in different pack sizes, only one entry was recorded. For each food product, the manufacturer, brand and product name, as well as the sodium content per 100 g were recorded. Where the product was owned and sold by the retailer only in their own store (private label products), the manufacturer name was taken as the name of the grocery retailer shown on the product [18]. Data were entered into The George Institute’s branded food composition database [19] according to standardized procedures [20]. Likewise, data were verified according to a defined quality assurance protocol and workflow, which includes screening for outliers and missing values, checking data entry accuracy by two study personnel independently and resolving queries and discrepancies by a review of the original NIP data, consultation between the research personnel, review of the manufacturer website or follow-up with the manufacturer directly.

### 2.4. Statistical Analysis

For bread and processed meats, the number and proportion of products with sodium content below the maximum FHD targets were calculated for each year. Pearson’s chi-squared test or Fisher’s exact test were used to assess whether the proportion of products meeting FHD targets differed between FHD participants and non-participants. We used the chi-squared test to determine whether the proportion of products meeting FHD targets were different between study baseline and 2013 for unmatched products, whereas the McNemar’s test was used for matched products that existed at baseline and in 2013. For breakfast cereals, products containing more than 400 mg sodium/100g in 2010–2012 were identified, and the change in the sodium content in these same products by 2013 was calculated and compared to the 15% FHD reduction target.

The mean, median and range of sodium content for products in each of the four food category targets were calculated in each year, overall and separately for FHD participants *vs*. non-participants and for individual food companies. We used linear mixed model to assess if the change in sodium content from baseline to 2013 was statistically significant and to determine whether the magnitude of the change differed between FHD participants and non-participants. Two-sided *p*-values of <0.05 were considered significant, and while there was no formal adjustment for multiple comparisons, the findings were interpreted in light of the number of comparisons made with the focus on the main comparisons and more extreme results. Statistical analyses were conducted using Stata 13.1 (Stata Corp, College Station, TX, USA).

## 3. Results 

### 3.1. Products and Food Companies Identified

Between 2010 and 2013, the sodium content of 1849 products was recorded. There were 885 bread products, 532 breakfast cereals, 387 bacon/ham/cured meats and 45 emulsified luncheon meats (Table 1). Due to the small number of emulsified luncheon meat products identified, subsequent analyses of these products were limited.

As expected, the majority of the products were supplied by FHD participants. For bread products, FHD participants included two branded food manufacturers (George Weston and Goodman Fielder) and three grocery retailers (ALDI, Coles, Woolworths) marketing their private label brands. For all years, more than half of the products from FHD participants were supplied by George Weston and Goodman Fielder (Table 1). For breakfast cereals, there were six FHD participants (ALDI, Coles, Kellogg, Nestle, Sanitarium and Woolworths) who together supplied 67% of breakfast cereal products in 2013. Approximately 30% of products in all years were Kellogg products. More than 88% of bacon/ham/cured meat products were supplied by FHD participants in all years, of which about a third were supplied by one manufacturer (Primo Smallgoods) (Table 1).

**Table 1:** The Food and Health Dialogue (FHD) targets and products included for: (**A**) bread; (**B**) processed meats (bacon/ham/cured meats); (**C**) processed meats (emulsified luncheon meats); (**D**) ready-to-eat breakfast cereals; for all FHD participants, all FHD non-participants products’ and individual FHD participants.

(A) Bread	2010	2011	2012	2013
	FHD target: maximum sodium 400 mg/100 g. Set in May, 2010, deadline to meet due by December, 2013. 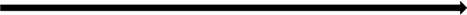			
FHD participants total products *n* (%)	145 (84%)	135 (63%)	146 (63%)	177 (66%)
ALDI	25	20	18	22
Coles	14	18	18	37
George Weston	45	36	41	46
Goodman Fielder	48	52	54	52
Woolworths	13	9	15	20
FHD non-participants total products *n* (%)	27 (16%)	79 (37%)	86 (37%)	90 (34%)
**(B) Bacon/ham/cured meat**	**2010**	**2011**	**2012**	**2013**
	FHD target: maximum sodium 1090 mg/100 g. Set in January, 2011, deadline to meet due by December, 2013. 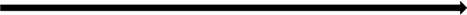			
FHD participants total products *n* (%)	83 (92%)	89 (89%)	81 (94%)	98 (88%)
ALDI	12	10	13	11
Coles	10	15	14	14
D’Orsogna	5	4	3	9
George Weston	12	17	14	17
Primo Smallgoods	37	36	27	37
Woolworths	7	7	10	10
FHD non-participants total products *n* (%)	7 (8%)	11 (11%)	5 (6%)	13 (12%)
**(C) Emulsified luncheon meats**	**2010**	**2011**	**2012**	**2013**
	FHD target: maximum sodium 830 mg/100 g. Set in January, 2011, deadline to meet due by December, 2013. 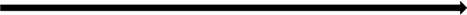			
FHD participants total products *n* (%)	9 (69%)	10 (67%)	5 (83%)	7 (64%)
ALDI	4	5	2	2
Coles	2	2	1	-
Primo Smallgoods	1	1	1	3
Woolworths	2	2	1	2
FHD non-participants total products *n* (%)	4 (31%)	5 (33%)	1 (17%)	4 (36%)
**(D) Breakfast cereals**	**2010**	**2011**	**2012**	**2013**
	FHD target: 15% less sodium if >400 mg/100 g. Set in May, 2010, and deadline to meet due by December, 2013. 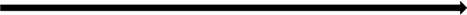			
FHD participants total products *n* (%)	86 (70%)	75 (69%)	97 (69%)	107 (67%)
ALDI	13	0	21	25
Coles	10	10	11	14
Kellogg	28	33	32	33
Nestle	17	19	19	21
Sanitarium	9	8	6	6
Woolworths	9	7	8	8
FHD non-participants total products *n* (%)	39 (30%)	33 (31%)	43 (31%)	52 (33%)

### 3.2. Proportion of Products Meeting FHD Targets

#### 3.2.1. Breads

**Figure 1:**
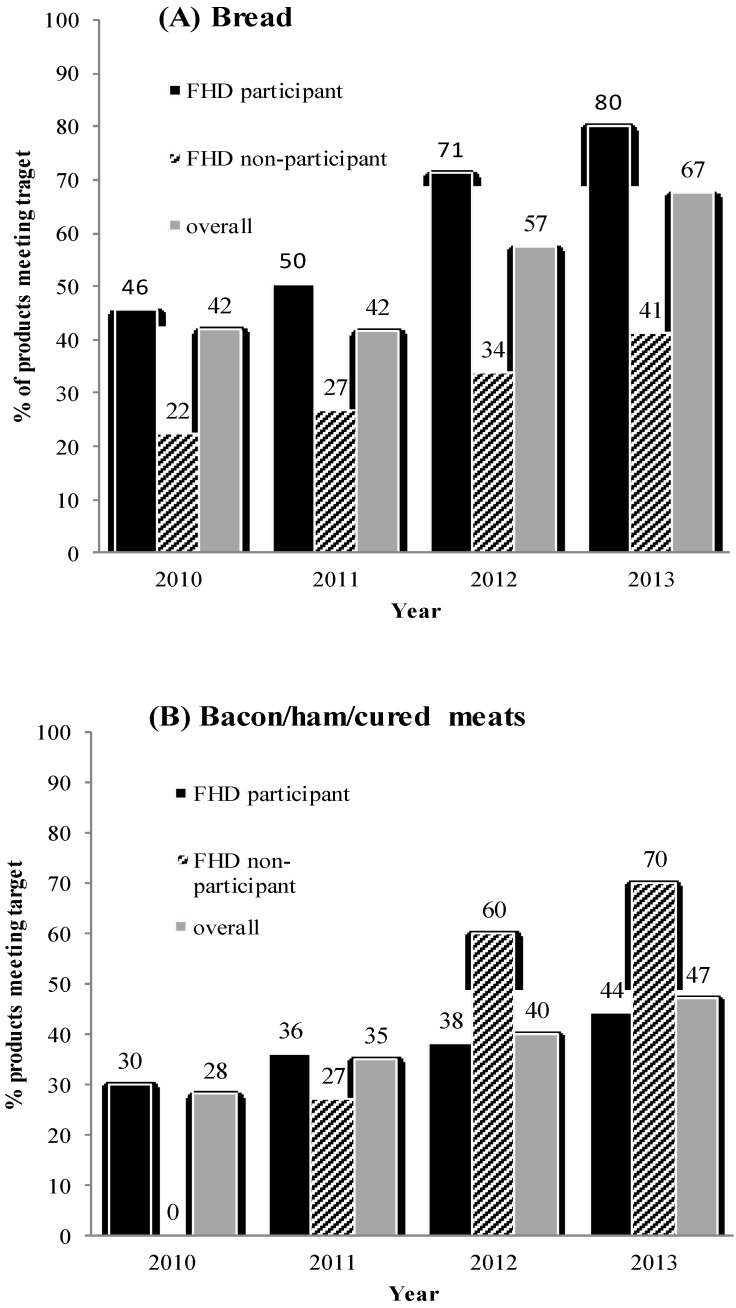
The proportion of products that met the respective Food and Health Dialogue (FHD) target by FHD participant status and overall for (**A**) bread, (**B**) bacon/ham/cured meats. For clarity, the exact percentages are shown for each bar.

**Figure 2:**
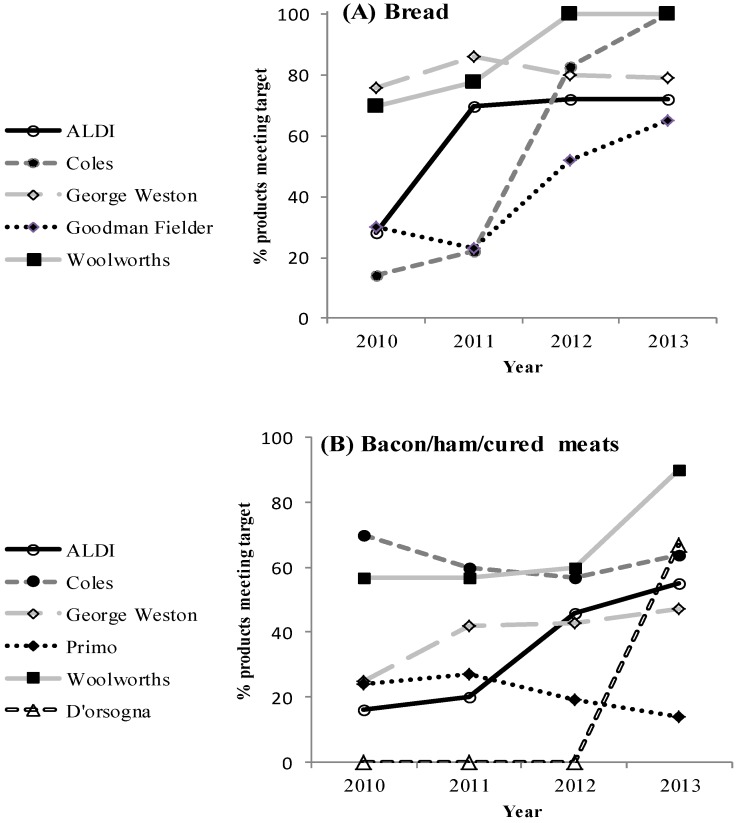
Proportion of products that met the respective Food and Health Dialogue (FHD) target by food companies participating in FHD from 2010 to 2013 for (**A**) bread, (**B**) bacon/ham/cured meats.

The proportion of all bread products meeting the FHD maximum target gradually increased from 42% in 2010 to 67% in 2013 (Figure 1A). The difference in the proportion of products meeting the targets in 2013 compared to 2010 was significant for unmatched products that were present in 2010 or 2013, but not both, (*n* = 327 products, chi-square test, *p* < 0.001), as well as for matched products that were available in all follow-up years (*n* = 55 products, McNemar’s test, *p* = 0.005). A greater percentage of FHD participant products met the target in both 2010 and 2013 compared to non-participants (*p* ≤ 0.02 for each, Figure 1A). The proportion of bread products meeting FHD targets for each of the FHD participants is shown in Figure 2A. There was substantial heterogeneity in the proportion of products meeting targets in 2013, as well as the rate of change for each company over time (*p* < 0.001). In 2013, compliance rates with FHD targets ranged from 65% to 100% for the five different companies. With the exception of George Weston Foods, which remained largely unchanged, the proportion of products meeting targets increased for the other four companies between 2010 and 2013 with 100% compliance by Coles (*n* = 37) and Woolworths (*n* = 20) in 2013. 

#### 3.2.2. Processed Meats

In 2010, 28% of all bacon/ham/cured meats met the FHD target, which increased to 47% by 2013 (Figure 1B). For unmatched products, 24% and 50% had a sodium level meeting the FHD target in 2010 and 2013, respectively (*p* = 0.001). There were 12 bacon/ham/cured meat products that had a sodium level recorded for all four years. Nine of these had a sodium content above the FHD maximum target in 2010, and just one had reformulated to meet the FHD target by 2013. Participant status did not influence the proportion of bacon/ham/cured meats meeting the target in 2013 (*p* = 0.14). For bacon/ham/cured meats, the proportion of products meeting sodium targets in 2013 also varied widely (14% to 90%) between the six participating companies (*p* = 0.002) (Figure 2B). Some companies increased the proportion of products meeting the FHD target (George Weston, ALDI, Woolworths and D’Orsogna), another showed little change (Coles) and Primo Smallgoods reduced the proportion of products meeting the target from 24% to 14% between 2010 and 2013.

#### 3.2.3. Ready-to-Eat Breakfast Cereals

Between 2010 and 2012, there were 25 products containing >400 mg/100 g sodium that were still available in 2013, and of these, 14 (56%) had reduced their sodium content by 15% or greater to meet the FHD target. The mean (range) reduction achieved was 21% (range 15%–26%). FHD participants had reduced sodium to meet the FHD target for 12 out of 19 products (63%) by 2013, whereas the non-participants achieved the sodium reduction target in two out of six products (33%) (*p* = 0.35). Of the 14 breakfast cereal products that had reduced sodium levels according to the FHD target, the majority (*n* = 10) were supplied by Kellogg. Of the 11 that had not had a reduction in sodium, six were supplied by ALDI.

### 3.3. Change in Mean Sodium Content for Each Product Category over Time

**Figure 3:**
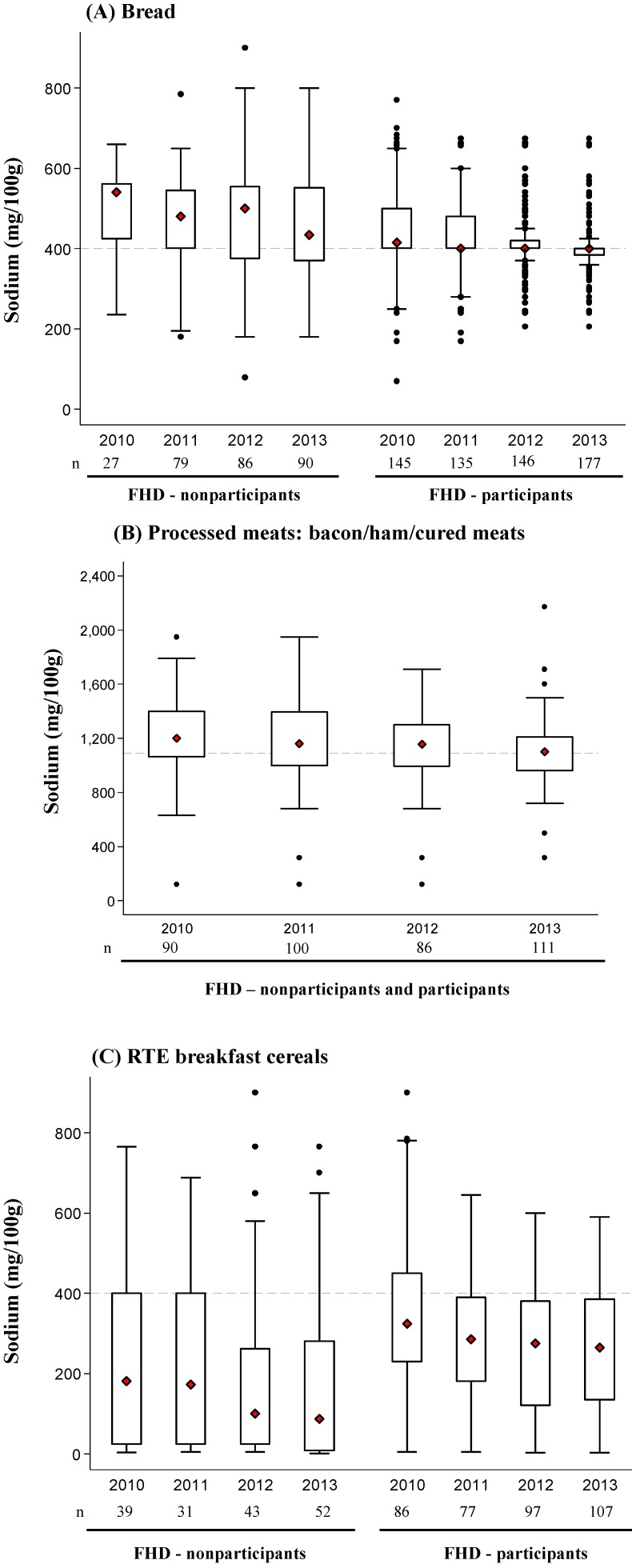
Distribution of sodium values from 2010 to 2013 for (**A**) bread, (**B**) ham/bacon/cured meats and (**C**) ready-to-eat (RTE) breakfast cereals. The box displays the interquartile range, and the median value is marked as a diamond. The lines extending above and below the box indicate the most extreme value within the 75th percentile + 1.5*x* (interquartile range) and the 25th percentile − 1.5*x* (interquartile range), and additional values outside of this range are marked as black circles. Values are shown separately for FHD participants and non-participants for bread and RTE cereal, but not processed meats, for which the great majority (~90% in each year) were supplied by FHD participants.

Figure 3 shows the distribution of mean sodium content for bread products, processed meats and breakfast cereals between 2010 and 2013. The mean sodium levels of bread products were 454 mg/100 g in 2010 (*n* = 172) and 415 mg/100 g in 2013 (*n* = 267), representing a 9% reduction (39 mg/100 g, *p* < 0.001). Sodium changes between 2010 and 2013 were similar for FHD participants (−47 mg/100 g) and non-participants (−53 mg/100 g) (*p_heterogeneity_* = 0.46). The sodium content of bread products remains highly variable, although for FHD participants, the variability declined from 2010 to 2013 and converged towards the target of 400 mg/100 g (Figure 3A). For bacon/ham/cured meat products, there was an 8% (101 mg/100 g) reduction in mean sodium content from 1215 to 1114 mg/100 g over the same time period (*p* = 0.001). This was true regardless of whether companies made a public commitment to the FHD targets or not (*p_heterogeneity_* = 0.18). Very few luncheon meat products were identified (<16 in each year). The mean sodium content of all emulsified luncheon meats fell from 980 mg/100 g to 886 mg/100 g (10% reduction), and five out of 11 products met the target in 2013.

Finally, for breakfast cereal products, the mean sodium content fell from 316 mg/100 g in 2010 to 237 mg/100 g in 2013 (25% reduction, *p* < 0.001). In 2010, the mean sodium level of breakfast cereals for FHD participants was greater compared to non-participants (114 mg/100 g, 95% CI, 36–192 mg/100 g, *p* = 0.005), and participants achieved slightly larger declines in mean sodium content over the study period (16 mg/100 g over three years, *p_heterogeneity_* = 0.03). There remained substantial heterogeneity in the sodium content of cereal products in 2013, with products from FHD participants’ having sodium levels ranging between 3 mg/100 g and 590 mg/100 g (Figure 3B). The maximum sodium content for products from FHD participants decreased from 900 mg/100g to 590 mg/100g between 2010 and 2013.

## 4. Discussion

These data provide the first objective evaluation of the changes in sodium content of bread, breakfast cereals and processed meats in Australia following the establishment of the FHD. Our results show that while excellent progress has been made by some food companies, overall voluntary reductions have not yet reached optimal levels. This is particularly so for processed meats, for which less than half of the products surveyed met the FHD 2013 targets. Our findings also identify substantial differences between the rates of product reformulation achieved by different food companies and argue for a more robust intervention strategy that is able to effectively engage with a larger proportion of the sector targeted.

Argentina and South Africa have introduced legislation regulating the maximum level of sodium that foods can contain [21], but the majority of countries with salt reduction targets have opted for a voluntary approach, as legislative processes are complicated and require considerable time to implement. The last decade in the UK. shows that a successful public private partnership (PPP) between government, industry and public health groups employing a multi-component strategy can lower population salt intake [16,22]. Setting sodium reduction targets and encouraging the food industry to gradually reformulate their products have been key elements of the UK. approach, which was initiated by the Food Standards Agency in 2003 and subsequently taken on by the Public Health Responsibility Deal for England in 2011. Progressively more challenging sodium reduction targets for over 60 food types have been set and, coupled with consumer campaigns and a threat of regulation, have achieved the most comprehensive ever falls in the sodium content of processed foods in a developed country, with reductions of up to 70% in some food categories reported [16,23,24,25]. The extent to which these methods can be transferred to other countries with different political and economic influences and consumer preferences has remained largely unknown. Our examination of the FHD program in Australia, which was based closely on the UK. approach, therefore provides novel evidence affirming the potential for a PPP target-based strategy to lower sodium content in other settings. The sodium reduction targets set by the FHD are closely aligned with the UK. targets for these three food categories.

Despite the progress made, our results also highlight areas of concern. The FHD participants negotiated, agreed and committed to take action on a series of modest, time-bound targets, but there was only partial voluntary compliance by the 2013 deadline for the first three categories identified. The wide range of sodium content observed in each of the food categories in 2013, including many products with sodium levels substantially below the FHD target, suggests technical capability is unlikely to be the major issue hindering compliance with the FHD targets. FHD participants did report technical and resource constraints hampering their efforts to reduce sodium in processed meats in November, 2012, but minutes from an FHD Executive Meeting in May, 2013, state that participants were nonetheless on track to meet targets in nominated products for December, 2013 [26]. The reason why there was a subsequent failure to voluntarily comply is therefore likely to be, in part at least, due to other shortcomings of the FHD program, such as the lack of monitoring and the absence of media publicity and public awareness of food company actions [16,27].

The marked difference in progress made by George Weston and Primo Smallgoods, two market leaders producing processed meats, is indicative of the problems that can arise with a weak PPP [28]. More products from George Weston met the target in 2013 compared to 2010, but the opposite was true for Primo Smallgoods. Heterogeneity in food reformulation efforts between manufacturers has also been reported in prior studies of sodium reduction [23,24,29,30,31], as well as for dietary fats [32]. It is beyond the scope of this study to assess what, and how, company characteristics influence action, but this is clearly an important area for future research given the central role that food reformulation will play in the prevention of diet-related ill health. Our observation of decreased sodium content in the products of both FHD participants and non-participants is encouraging and suggests that engagement with the major food manufacturers and grocery retailers may produce flow-on effects in companies other than those directly engaged. 

The sodium in food is essential for the taste characteristics of many products and plays other functional roles in manufacture and storage. For example, in bread, sodium controls yeast activity and aids in processing; in breakfast cereals, it enhances texture, and in some processed meats, it acts as a preservative by preventing the growth of microorganisms and, thus, increasing shelf life [33,34]. Efforts to reduce sodium content will therefore be facilitated by technological innovation; although this is a more pressing requirement for some food categories than others, better and more widespread application of existing technology still appears to present a significant opportunity [33].

Excess dietary salt drives the rise in blood pressure with age, and population-wide salt reduction has been identified as a priority public health goal for the next decade by the World Health Organization in its efforts to avert chronic disease [35]. Breads, breakfast cereals and processed meats were prioritised for sodium reduction by the FHD, as they are frequently consumed by a large portion of the Australian population [17]. The 2011–2012 National Nutrition and Physical Activity Survey found that in the 24 h prior to interviewing, two thirds of Australians had eaten two slices of regular bread, more than a third had eaten breakfast cereals and almost a quarter had consumed processed meats [13]. Together, these three food categories were estimated to be responsible for about one fifth of daily salt intake in adults, making them a good initial target for salt reduction efforts [13]. However, if effects comparable to those in the UK. are to be achieved [22], there will need to be an expansion of the FHD program to cover additional food categories, further lowering of the existing category targets and new efforts to encourage better industry voluntary compliance. Given that mean salt intake among Australians is currently some two-fold higher than the upper level suggested [3,5,36], learning the lessons of the UK. program should be a priority [25]. 

A key strength of our study is the consistent and repeated sampling method used to obtain sodium data on a comprehensive range of breads, breakfast cereals and processed meat products from grocery retailers in Australia, who together have about 95% of the market share of packaged groceries [37]. Data tracking over sequential years allowed for an assessment of the reformulation of foods that were available in multiple years, as well as an examination of the sodium level of new products launched each year. Results were similar for all products and for products that were available in multiple years, which suggest progress towards FHD targets were met via both reformulation, as well as the introduction of new lower sodium options. Whereas prior monitoring studies generally focused on single food categories [24,30,31], we expanded our analyses to include multiple product categories that are major contributors of dietary sodium in Australia and highlighted the substantially different progress made for each category. Finally, we carefully tracked products from individual food companies and retailers to contrast and compare their efforts.

Our study also had several weaknesses. Coverage of foods in the categories we studied is unlikely to be complete. Due to the small number of emulsified luncheon meat products identified in all years, there was a high level of uncertainty in these results. Therefore, our findings should be interpreted with caution. We were unable to include breads from in-store bakeries or processed meats from in-store delicatessens, as these products do not carry a NIP from which to collect data and are therefore excluded. Nonetheless, data collection included private label and branded products and is likely to be broadly representative of packaged foods available for sale in Australian supermarkets. We were unable to assess all of the food companies involved in the FHD [17], as the products of some do not appear to be sold directly to the consumer through the supermarkets we sampled (e.g., they supply ingredients for food service). The integrity of our nutritional data also depends on the accuracy of the NIP and relies on food companies to update the NIP as products are reformulated. Prior studies suggest that NIP data in Australia is generally accurate and reliable [38].

## 5. Conclusions 

Food manufacturers have made moderate progress towards meeting the FHD sodium targets for bread, breakfast cereal and processed meats, leading to modest overall reductions in the mean sodium levels in these food categories. The food companies that have made these changes should be applauded for their efforts to improve public health nutrition in Australia. Nevertheless, progress towards meeting the FHD goals remains incomplete and differed substantially between food companies. Our results highlight the importance of additional work to determine why some companies took the opportunity to act and reformulate, while others supplying similar food types did not. Our data also argue for additional systematic evaluation of interim and scheduled targets for other food categories being addressed by the FHD in conjunction with a review of the FHD governance process to determine whether the current public-private partnership model is fit-for-purpose. A sustained small to-moderate reduction in sodium content in processed foods will likely reap significant public health gains, so it is important that food companies continue to work towards the FHD sodium reduction targets and are monitored closely against their progress.
